# Minimally Invasive Aortic Valve Replacement in Osteogenesis Imperfecta: A Case Report

**DOI:** 10.70352/scrj.cr.25-0469

**Published:** 2025-10-02

**Authors:** Noriyuki Takashima, Chihiro Yokoyama, Taku Tanaka, Suguru Matsui, Bruno Yuji Chimada, Shintaro Okuda, Yuji Matsubayashi, Fumihiro Miyashita, Tomoaki Suzuki

**Affiliations:** Division of Cardiovascular Surgery, Department of Surgery, Shiga University of Medical Science, Otsu, Shiga, Japan

**Keywords:** osteogenesis imperfecta, right anterior mini-thoracotomy, aortic regurgitation, minimally invasive cardiac surgery

## Abstract

**INTRODUCTION:**

Cardiac surgery in patients with osteogenesis imperfecta is a high-risk procedure because of tissue fragility, which leads to complications, such as bleeding, fractures, and increased mortality. A less invasive approach, such as right anterior mini-thoracotomy, may help reduce these risks and improve outcomes.

**CASE PRESENTATION:**

A 36-year-old woman with severe aortic regurgitation was referred for surgery. She had type I osteogenesis imperfecta, which was diagnosed based on childhood fractures, deafness, and a family history of the disease. To avoid sternotomy and minimize the risk of fracture, aortic valve replacement was performed through a small right anterior thoracotomy using a wound retractor instead of a thoracotomy device. A 23-mm mechanical valve was implanted with cross-clamp and cardiopulmonary bypass times of 62 and 107 min, respectively. The patient’s postoperative course was uneventful. At the 2-year follow-up, she remained in good condition, with no significant echocardiographic findings.

**CONCLUSIONS:**

For patients with osteogenesis imperfecta, the right anterior mini-thoracotomy approach is an excellent surgical option that may help improve outcomes.

## Abbreviations

AR
: aortic regurgitation
AVR
: aortic valve replacement
OI
: osteogenesis imperfecta

## INTRODUCTION

OI is a connective tissue disorder caused by a genetic abnormality in type I collagen and is sometimes complicated by valvular heart diseases, such as AR.^1)^ Cardiac surgery in patients with OI is associated with a high rate of complications, including bleeding and fractures owing to tissue fragility, resulting in a high mortality rate.^2–4)^ Approaches through a right anterior mini-thoracotomy may reduce such complications and help improve surgical outcomes.

## CASE PRESENTATION

A 36-year-old woman with severe AR was referred to our outpatient clinic. The patient complained of fatigue during exertion. She was diagnosed with type I OI based on her history of deafness and 3 childhood fractures. The patient’s mother, grandmother, and 2 children were also diagnosed with type I OI.

**Fig. 1:**
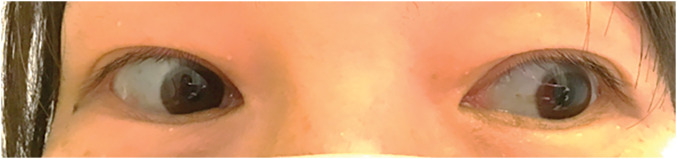
The image shows bilateral blue discoloration of the sclerae, a hallmark clinical feature of osteogenesis imperfecta.

**Fig. 2:**
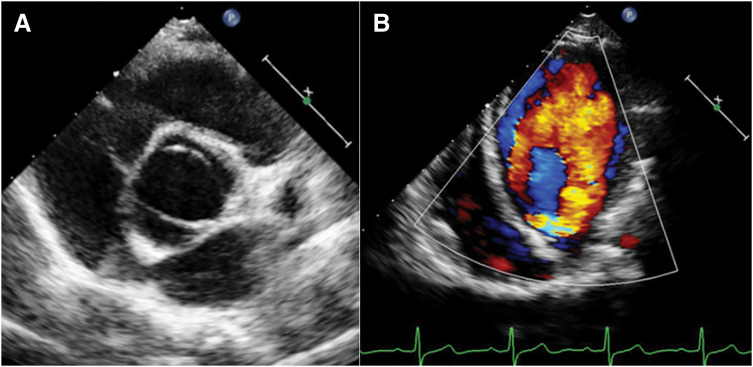
Preoperative echocardiography findings. (**A**) Short-axis view showing the bicuspid aortic valve. (**B**) Four-chamber view showing a severe aortic regurgitation jet swirling within the left ventricle.

Physical examination revealed blue sclerae (**Fig. 1**) and a diastolic murmur on auscultation, but no signs of heart failure or pulmonary congestion. Echocardiography showed severe AR with a bicuspid valve and a pressure half-time of 218 ms; the left ventricular ejection fraction was 58% (**Fig. 2**). The patient showed no signs of coronary artery disease or aortitis.

**Fig. 3:**
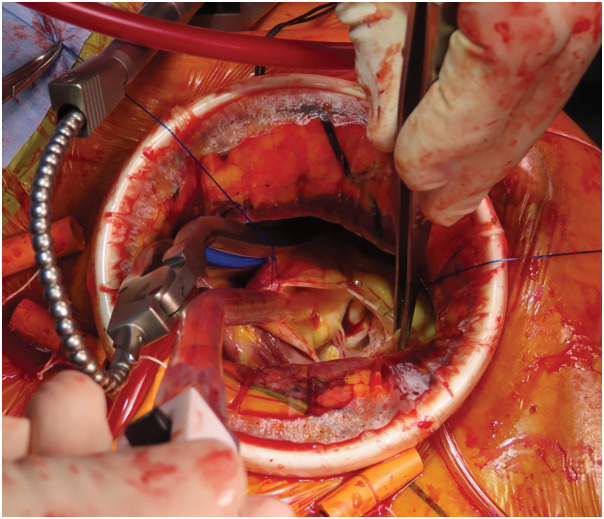
Intraoperative view of the surgical field. A good surgical field was achieved using only a wound retractor.

To avoid sternotomy, we performed AVR via right anterior mini-thoracotomy through the 4th intercostal space using a 6-cm skin incision. To avoid fractures, a wound retractor (Alexis; Applied Medical, Rancho Santa Margarita, CA, USA) was used instead of a thoracotomy device, which provided a good surgical field (**Fig. 3**). Cardiopulmonary bypass was established using femorofemoral arteriovenous cannulation, and cold cardioplegia was selectively infused into each coronary artery after aortic clamping. The aortic valve was bicuspid and prolapsed into the left ventricle. The valve was resected, and a 23-mm mechanical valve (SJM Regent; Abbott, Abbott Park, IL, USA) was implanted in an intra-annular position. We performed the procedure using standard techniques, with emphasis on the preservation of sufficient annular tissue to prevent valve dehiscence. The cross-clamp and cardiopulmonary bypass times were 62 and 107 min, respectively.

The patient’s postoperative course was uneventful, and she was discharged on POD 7. At the 2-year follow-up visit, she was in good physical condition and repeat echocardiography showed no significant abnormalities.

## DISCUSSION

OI is a congenital disorder that presents with varying degrees of connective tissue symptoms, in addition to an increased tendency for fractures and progressive bone deformity due to generalized bone fragility. More than 90% of OI cases are caused by qualitative or quantitative abnormalities in type I collagen (COL1A1 and COL1A2) owing to genetic mutations. The inheritance pattern is either autosomal dominant or recessive.

Patients with OI may develop heart failure due to valvular heart disease, with AR being particularly common. A Norwegian study of 99 patients aged ≥25 years with OI reported an AR prevalence of 20.2% (mild AR in 10.1% and moderate or greater severity in 10.1%).^1)^ In an Italian study of 40 patients with OI without heart failure symptoms aged ≥19 years, the reported AR prevalence was 40%, even in the absence of valvular structural deformity.^5)^ Therefore, it is recommended that patients with OI undergo periodic echocardiographic evaluations once they reach adulthood.

In patients with OI undergoing cardiac surgery, the operative mortality rate is reportedly high, ranging from 21% to 50%.^2–4)^ The selection of prosthetic valves for patients with OI is also critical. In a study by Dimitrakakis et al.,^6)^ patients receiving mechanical valves had higher operative mortality and complication rates than those receiving bioprosthetic valves, although the difference was not significant. Therefore, bioprosthetic valves were recommended. However, long-term survival rates of 15^7)^ and 18 years^8)^ have been reported in patients receiving mechanical valves. As most patients with OI are relatively young, the choice of prosthetic valves should be individualized based on patient preferences.

In this case, after thorough discussion with the patient, a mechanical valve was chosen for its superior durability. We also carefully considered the use of warfarin, particularly regarding bleeding complications. However, in patients with OI, the bleeding tendency is thought to result from platelet dysfunction and capillary fragility, which are difficult to detect in preoperative evaluations.^9,10)^ A history of bleeding in patients with OI is a known risk factor for postoperative hemorrhage, and in such cases, mechanical valves should be avoided.^10)^ Naturally, we also consider the potential effects of warfarin therapy and maintain close cardiology follow-up even after discharge.

Complications, such as bleeding, fractures, and bone disjunction, have also been reported. Therefore, a right anterior mini-thoracotomy approach that does not require a sternotomy, as in the present case, is extremely useful.^11)^ Because our patient had a small stature, we were able to achieve an adequate surgical field using only a wound retractor, thereby avoiding the need for a thoracotomy device. As minimally invasive cardiac surgery becomes more widespread, improved surgical outcomes in patients with OI can be anticipated.

## CONCLUSIONS

For patients with osteogenesis imperfecta, a right anterior mini-thoracotomy is an excellent surgical option and may help improve outcomes.

## DECLARATIONS

### Availability of data and materials

The authors have full control over all primary data and agree to allow the journal to review their data if requested.
